# Imminent Respiratory Collapse in Advanced Head and Neck Cancer: A Case Report and Discussion

**DOI:** 10.1177/10966218251368266

**Published:** 2025-08-21

**Authors:** Molly Svendsen, Mark Edwin

**Affiliations:** Division of Palliative Medicine, Mayo Clinic Arizona, Phoenix, Arizona, USA.

**Keywords:** carotid blowout syndrome, imminent respiratory collapse, squamous cell cancer

## Abstract

Airway obstruction is a distressing and potentially life-threatening complication in patients with advanced head and neck cancers, particularly squamous cell carcinoma (SCC) of the pharynx. This case highlights the clinical, ethical, and interdisciplinary complexities involved in managing airway compromise in the context of progressive disease and limited treatment options. A 75-year-old man with recurrent SCC of the soft palate, nasopharynx, oropharynx, and hypopharynx, recently initiated on pembrolizumab and radiation therapy, presented with dysphagia, stridor, and intermittent tumor bleeding. Imaging revealed a large pharyngeal mass causing critical airway narrowing. Plans for tracheostomy to manage anticipated radiation-induced edema were aborted following the patient’s acute neurological deterioration, with imaging confirming bilateral carotid artery occlusions and a new stroke. With poor prognosis and elevated risk of carotid blowout syndrome (CBS), further disease-directed treatments were deemed inappropriate. A multidisciplinary team, including palliative care, oncology, and ENT, facilitated a transition to comfort-focused care, emphasizing symptom control, airway management, and emotional support. The patient was transferred to inpatient hospice for end-of-life care. This case underscores the multifactorial nature of airway obstruction in advanced pharyngeal SCC, including tumor burden, treatment-related edema, and vascular complications. It highlights the importance of risk stratification for tracheostomy, especially in the setting of stroke, bleeding risk, and impending CBS. Symptom management was pivotal in maintaining comfort. Nonpharmacologic strategies and psychosocial-spiritual support further contributed to comprehensive end-of-life care. Individualized, multidisciplinary, and patient-centered approaches are essential when curative therapies are no longer appropriate in advanced head and neck cancer. Prioritizing comfort, clear communication, and integrative symptom management can optimize quality of life and support dignity at the end of life.

## Introduction

Airway obstruction is a significant and potentially life-threatening complication in patients with advanced head and neck squamous cell carcinoma (HNSCC), particularly when tumors involve the pharyngeal region. The progression of malignancy, combined with treatment-related effects such as edema from chemoradiation, can lead to critical airway narrowing, necessitating prompt and effective management strategies. Tracheostomy has traditionally been employed to secure the airway in such scenarios; however, its implementation must be carefully considered against potential risks, including procedural complications and the patient’s overall prognosis.

The decision-making process becomes even more complex in cases where patients present with additional comorbidities or complications, such as vascular involvement or neurological deficits. In these situations, the benefits of invasive interventions like tracheostomy must be weighed against the likelihood of improved outcomes and quality of life. Palliative care principles play a crucial role in guiding treatment decisions, focusing on symptom management and aligning care with the patient’s goals and values.

This article presents a case study of a 75-year-old man with recurrent SCC of the soft palate, nasopharynx, oropharynx, and hypopharynx, who developed significant airway obstruction during treatment. The case underscores the complexities involved in managing airway compromise in advanced head and neck cancer and highlights the importance of individualized, multidisciplinary approaches that prioritize patient-centered care and quality of life considerations.

## Case Description

A 75-year-old man with a history of recurrent squamous cell carcinoma (SCC) of the soft palate, nasopharynx, oropharynx, and hypopharynx, who had recently begun radiation therapy and treatment with pembrolizumab, presented with weakness, dysphagia, and intermittent epistaxis. Given his dysphagia, he had recently undergone gastrostomy tube placement for nutritional supplementation. At baseline, he was functionally independent but limited.

On arrival to the hospital, he was afebrile and hemodynamically stable on room air but exhibited significant stridor, indicating potential airway compromise. He was noted to have moderate anemia and lactic acidosis. A CT head scan showed growth of his oral cavity mass measuring approximately 3.4 cm transverse by 2.6 cm AP by 4.2 cm CC at the largest cross-section causing significantly narrowing the airway. He experienced intermittent mild bleeding from the tumor site, requiring suctioning.

The patient was subsequently admitted to the hospital and received a unit of packed red blood cells, which stabilized his hemoglobin and normalized his lactate. Given the risk of airway obstruction and bleeding, ENT attempted to perform a transnasal laryngoscopy but could not pass the scope beyond the nasopharynx due to the tumor. Intraorally the tumor was noted to occupy approximately 80–90% of the oropharynx. ENT expressed concern that radiation-induced laryngeal edema could further compromise the airway, worsening stridor and potentially cause complete obstruction. Therefore, a tracheostomy was recommended before resuming radiation to improve airway control during treatment and reduce airway-related complications.

On the day scheduled for tracheostomy, the patient developed acute-onset rightward gaze deviation and left-sided neglect. CT imaging of the head revealed bilateral internal carotid artery occlusions—one chronic, one new. A decision to forgo the tracheostomy was made given the patient’s poor prognosis and the risk of further tumor bleeding, which outweighed the procedure’s potential benefits. Furthermore, anticoagulation for his stroke posed a significant risk given the bleeding tumor and precarious airway.

After foregoing the tracheostomy, oncology determined the patient was no longer a candidate for disease-directed treatments. The risks of radiation therapy-induced airway compromise, infection, and bleeding, coupled with the progression of his disease, suggested that aggressive treatment would likely cause more harm than benefit.

After discussions with the patient, his family, and the multidisciplinary team, comfort-focused care was prioritized. The Palliative Medicine team, collaborating with oncology, adjusted the care plan to optimize symptom management, focusing on pain relief, airway management, and bleeding control. The prognosis was clarified for the patient. He was subsequently transferred to an inpatient hospice facility for end-of-life care.

## Discussion

Airway obstruction can be a distressing complication in patients with advanced oral cavity SCC.^1^ Studies indicate that up to 27.8% of head and neck cancer patients experience airway-related complications requiring tracheostomy due to respiratory failure.^2^ The primary causes are multifaceted, stemming from tumor growth, treatment-related complications, and infections. Tumor progression, particularly in the pharynx, directly obstructs critical airway structures (larynx and trachea), leading to dyspnea and respiratory distress.^2^ The proximity of pharyngeal tumors to the upper airway exacerbates this, increasing the risk of complete airway collapse.^3^ Several key risk factors significantly increase the likelihood of airway obstruction. Pretreatment dysphagia impairs the ability to clear secretions and swallow effectively, leading to increased risk of aspiration and progressive airway compromise.^2,4^ Concurrent chemoradiation can further exacerbate the risk by causing mucositis, edema, and fibrosis, worsening airway obstruction.^5^ Beyond these treatment-related and preexisting conditions, additional risk factors contribute to poorer outcomes and increased need for intervention, including smoking, alcohol use, male sex, and older age.^6–8^ Larger tumors also tend to cause more significant local invasion, further complicating airway management.^9^ The tumor’s location is crucial; oropharyngeal and hypopharyngeal tumors are particularly prone to causing obstruction due to their proximity to critical airway structures.^9^

The primary symptom experienced by patients with advanced oral cavity SCC and airway obstruction is dyspnea, reflecting airway compromise. Severity ranges from mild discomfort to life-threatening respiratory failure. Treatment modalities vary depending on severity and the patient’s overall condition. For severe cases, tracheostomy may be necessary to secure the airway, providing a direct route for ventilation and suctioning of secretions.^10^ This is particularly considered when radiation-induced edema is anticipated to further compromise the airway.^5,10^ However, the decision to proceed with a tracheostomy involves careful consideration of the patient’s overall health and prognosis. In addition to directly addressing airway obstruction, symptom management includes pharmacologic interventions such as opioids for dyspnea^11,12^ and benzodiazepines for anxiety.^13^

Opioids are commonly used to manage dyspnea in patients with advanced cancer and airway obstruction by reducing the sensation of breathlessness by altering the central perception of dyspnea and reducing the ventilatory response to hypoxia and hypercapnia.^11–13^

Benzodiazepines, such as lorazepam and diazepam, are used primarily to address the anxiety component associated with dyspnea.^14^ A systematic review and meta-analysis indicated that while benzodiazepines alone do not significantly improve dyspnea compared to opioids alone, the combination of benzodiazepines and opioids may be more effective.^14^

However, the concurrent use of opioids and benzodiazepines has also been associated with an increased risk of respiratory depression, overdose, and mortality, particularly in medically complex or frail patients. This is due to the synergistic sedative effects of both drug classes, which can suppress central respiratory drive.^15^ Clinicians should carefully evaluate the risks and benefits of combination therapy, especially in patients with compromised pulmonary status, and initiate treatment at the lowest effective doses with close monitoring. When used thoughtfully, these agents can provide meaningful relief, but their combined use should be individualized and closely monitored.

Anticholinergic medications, such as scopolamine, glycopyrrolate, and atropine, are commonly used as adjuncts to manage dyspnea in patients with oropharyngeal SCC, particularly when excessive oropharyngeal secretions contribute to noisy breathing or the sensation of dyspnea.^12,16^ These agents work by reducing secretion production, which can be beneficial for patients who are unable to clear secretions effectively due to tumor burden or neuromuscular dysfunction. A randomized clinical trial demonstrated that prophylactic subcutaneous scopolamine butylbromide decreased the incidence of death rattle at the end of life, supporting its role in symptom control.^16^ However, a subsequent Cochrane review demonstrated that anticholinergics were as effective as a placebo in decreasing audible respiratory secretions at end-of-life.^17^ In addition, anticholinergic medications carry potential risks, including dry mouth, urinary retention, constipation, blurred vision, cognitive impairment, and delirium—side effects that can be especially burdensome in patients already experiencing significant symptom burden and reduced cognitive reserve. Thus, these medications are contraindicated in patients with delirium, significant cognitive impairment, or urinary retention. Additional caution should be taken when patients are already on medications with anticholinergic properties, including first-generation antihistamines, antimuscarinic agents, tricyclic antidepressants, and antispasmodics.^18^ In addition to pharmacologic interventions, nonpharmacologic strategies such as positioning to optimize respiratory function and regular suctioning to clear airway secretions should be incorporated into the management plan to further support patient comfort and alleviate symptoms of dyspnea.^17^ Specifically, positioning the patient with the head of the bed elevated at 30 to 45 degrees facilitates postural drainage, maximizes diaphragmatic excursion, and helps maintain airway patency. Repositioning should occur every two to four hours or more frequently as tolerated to prevent secretion pooling.^17^ For patients with copious oropharyngeal secretions, gentle oropharyngeal suctioning should be performed as needed, rather than on a scheduled basis, to minimize discomfort, gagging, or mucosal trauma.^17^ Frequent reassessment of secretion burden and distress level should guide suctioning intervals, with the goal of reducing audible secretions and improving comfort without causing agitation or distress. In our patient’s case, carotid artery occlusion raised concern for carotid blowout syndrome (CBS), a life-threatening complication in HNSCC. CBS results from arterial erosion or rupture due to tumor invasion, prior chemoradiation, or surgery.^19^ Risk factors include advanced tumor stage (T4b), carotid encasement, local recurrence, chemoradiation (HR ∼2.0), radical neck dissection, and high-dose radiation (≥70 Gy)^19^ Clinical and radiographical signs of impending CBS include headache, exposed carotid on nasoendoscopy, skull base erosion, and air-containing necrosis.^19^

An essential turning point in this case was the decision to involve the palliative medicine team and transition the patient to hospice care. This reflected a robust multidisciplinary approach to individualized care, with contributions from oncology, ENT, and palliative care working in close coordination. Oncology provided prognosis clarification and guidance regarding the infeasibility of further disease-modifying therapies and evolving airway risk. ENT offered critical insight into the anatomical extent of the tumor, procedural risks, and likelihood of airway compromise. The interdisciplinary palliative medicine team led goals-of-care discussions, elicited the patient’s values, and managed complex symptoms, supported the patients spiritual needs through daily prayer and guided meditation and assisted in navigating the logistics for hospice transition. In summary, this case illustrates the importance of individualized, patient-centered care in managing complex conditions like SCC of the pharynx. The integration of pharmacologic and nonpharmacologic treatments, as well as effective communication about prognosis and care goals, is essential in improving quality of life for patients facing the end of life.

## Statement of Informed Consent

Informed consent was obtained from the patient involved in this case report.
